# A Symposium on the Clinic of the Future and Telehealth: Highlights and Future Directions

**DOI:** 10.7759/cureus.15234

**Published:** 2021-05-25

**Authors:** Austin B Carpenter, Evan Sheppard, Shireen Atabaki, Natasha Shur, Annie Tigranyan, Theresa Benchoff, Adam Snyder, Aliza Fisher, Kevin Cleary

**Affiliations:** 1 Neurological Surgery, Georgetown University Medical Center, Georgetown, USA; 2 Orthopedics and Pediatrics, Sheikh Zayed Institute for Pediatric Surgical Innovation, Children's National Health System, Washington, USA; 3 Telemedicine and Emergency Medicine, Children's National Health System, Washington, USA; 4 Genetics and Metabolism, Rare Disease Institute, Children's National Health System, Washington, USA; 5 Family Medicine, Premier Primary Care, Arlington, USA; 6 Anatomy, George Washington University, Washington, USA; 7 Medicine, Yale College, Yale University, New Haven, USA; 8 Medical Device Development, Sheikh Zayed Institute for Pediatric Surgical Innovation, Children's National Health System, Washington, USA

**Keywords:** clinic of the future, telemedicine

## Abstract

Children’s National Hospital held a virtual symposium on “The Clinic of the Future and Telehealth” in December 2020. The goal of the symposium was to explore future trends in these domains. We also discussed how the coronavirus disease 2019 (COVID-19) pandemic accelerated ongoing changes in healthcare. We explored what is on the horizon in these fields and how these changes might affect care delivery in the future. Specifically, we discussed the “Clinic of the Future” with clinical teams from genetics and metabolism, orthopedic surgery, and primary care while our telehealth discussion involved genetics and metabolism, psychiatry, and telerehabilitation. As one example, wearable technology could be adopted among primary care practices and drive a shift in outpatient care from center-based care to patient-based care. We also examined technological innovations in physical exam instruments, gait analysis, imaging integration, and cast technology that could modernize the orthopedic surgery clinic. Telemedicine has rapidly expanded among all fields of medicine, especially since the COVID-19 pandemic, and has spurred innovation to improve the effectiveness of virtual physician visits. The development of technology to improve the virtual physical exam, during a telemedicine visit, further increases the utility of online appointments and increases access to care in all specialties. The incorporation of photogrammetry technology, in genetics and metabolism dysmorphology exams, will offer standardized tracking of patients that could improve diagnosis and treatment. Psychiatry has found nearly equal efficacy in diagnosis and treatment with telehealth visits and the additional benefit of gaining insight in the setting of the patients’ home. Robotics has become increasingly common in rehabilitation, which can now incorporate a gaming experience that can be remotely updated and increase engagement and adherence in pediatric patients. The continued exploration of new ideas promises to improve both in-person and virtual care options.

## Introduction

In this paper, we summarize the results of a virtual symposium held on telehealth and the clinic of the future at Children’s National Hospital in Washington, DC in December of 2020. The goal of the “Symposium on the Clinic of the Future and Telehealth” was to explore the future trends of ambulatory clinical care and telehealth as well as to discuss how the coronavirus disease 2019 (COVID-19) pandemic has accelerated these changes. Here, we explore what is on the horizon in these fields and how these changes may affect the delivery of care in the future.

The unprecedented conditions arising from the COVID-19 pandemic have rapidly brought on changes to healthcare delivery and are further accelerating change toward the clinic of the future [1]. Many avenues of medical care have been revolutionized in recent months, especially in the delivery of ambulatory medicine. While the use of telemedicine has increased in recent months, there are many areas of the ambulatory visit that would benefit from innovation in workflow, exam room design, physical exam tools, electronic medical record (EMR) integration, ancillary services, and informatics [2].

The ongoing pandemic has had unprecedented effects on all aspects of medical care and the global healthcare system [3]. It has stimulated a rapid increase in Internet and telecommunication-based medical services previously underutilized by many medical departments [3]. Telemedicine has been defined as the use of telecommunication and information technologies to support healthcare delivery remotely [4]. Telehealth is a broader term that includes telemedicine, the technology used to provide access to patient data, transfer clinical data, diagnose, and provide treatment interventions [4-5]. The trend of increased use of telemedicine began before the COVID-19 pandemic in the United States but was accelerated by the pandemic [5]. From 2010-2017, the percentage of US hospitals that used video technologies to connect to patients increased from 35% to 76%, and this has only further increased during the pandemic [5].

Here, we first discuss telemedicine with a focus on its benefits and limitations. We review unique aspects of its adoption in genetics and metabolism, psychiatry, and rehabilitation. We investigate the impact current telemedicine practices will have on modern medical practice and the clinic of the future. We follow this discussion by further exploring technologies that could be adopted for the clinic of the future. We explore the virtual physical exam, wearable technology, and the clinic of the future in orthopedic surgery. We then finish with discussion and conclusions to provide further insight and future directions. We are creating a website with the symposium contents, which will be available at the time of publication of this paper.

## Technical report

**Figure 1:**
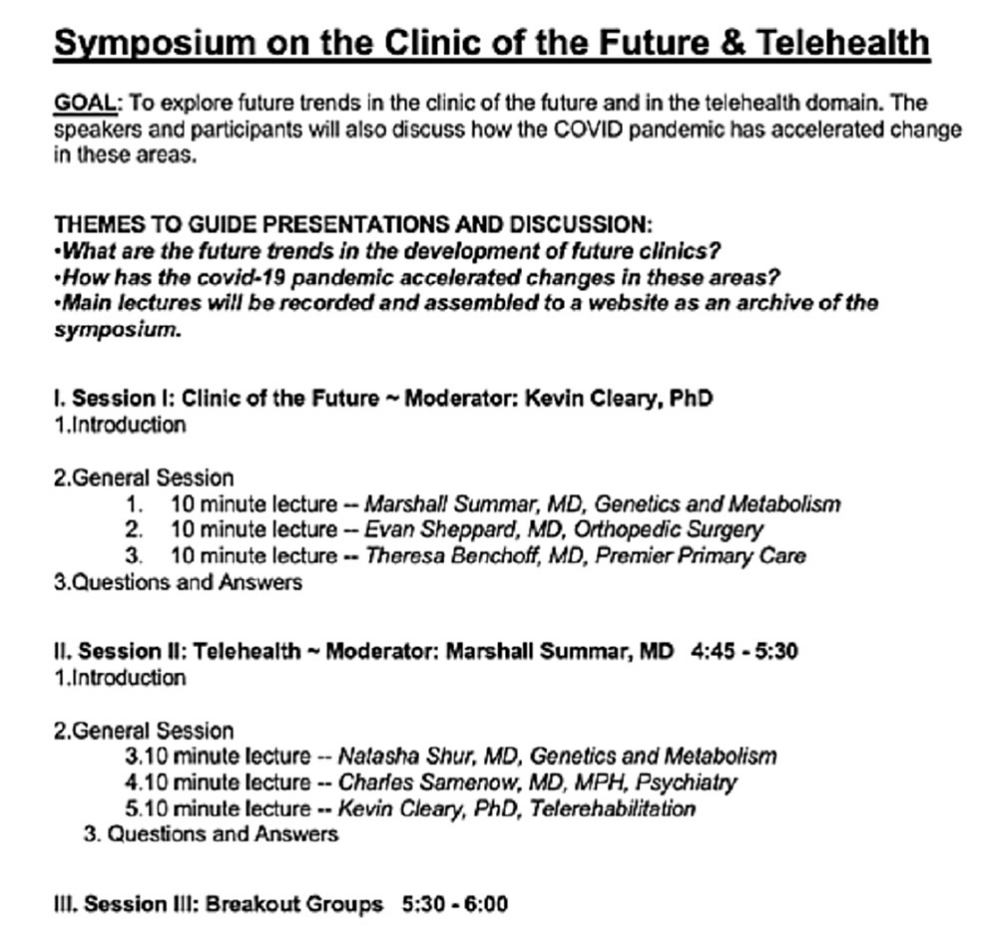
Agenda of the Symposium on the Clinic of the Future and Telehealth hosted by Children’s National Hospital on Thursday, December 10, 2020, 4-6 pm

To discuss the role of telehealth in modern practice, three speakers from genetics and metabolism, psychiatry, and telerehabilitation reviewed the use of virtual medicine in their practices. The clinic of the future was discussed with three attending physicians from genetics and metabolism, orthopedic surgery, and family medicine who spoke on the technical developments in their respective fields and the use of emerging technology. The symposium agenda is highlighted in Figure 1. Here, we summarize our symposium and document pertinent findings.

Telehealth

Telehealth in Genetics and Metabolism

Telemedicine in clinical genetics and metabolism offers advantages in assessing patients, especially in the pediatric population where patients remain comfortable in their own homes and within their natural environments. Valid observations can thus be made regarding the patient function that otherwise may have been hampered or obscured by a busier clinical setting foreign to the patient [6]. This was particularly reported among patients with autism who felt more comfortable in their home surroundings [6]. These digital appointments also allow families with medically fragile children to avoid the burden and risks of transportation.

While satisfaction among patients and physicians is high in this group, several limitations were recognized regarding genetics telemedicine examinations, including lack of hands-on measurements, physical exam, and poor image quality, especially for careful inspection of the skin [6]. Physical measurements of pediatric patients are necessary for a genetics and metabolism evaluation, particularly to plot growth curves, and a lack of standard methods among patients is a shortcoming of digital appointments. Likewise, a complete physical exam is also difficult to perform virtually. Patients without measuring tapes have used household items with known dimensions like dollar bills; however, a reliable exam remains elusive via telemedicine. Image technology has been proposed to address this problem of taking measurements of pediatric patients by using existing 3D Photogrammetry technology from other industries and applying it to medicine. This technology would allow photographs and videos to be taken of the patient and would compile this data along with height, weight, and head circumference into a three-dimensional model. Embedded software would then create a 3D rendition of the pediatric patient to follow changes in morphology over time. This would significantly benefit the dysmorphology exam, which could then allow for better visualization of current diagnoses, turn a subjective assessment into a reproducible objective assessment, aid in treatment planning, and ultimately improve physician-patient communication.

Telehealth in Psychiatry

Psychiatry is one of the medical fields most amenable to telemedicine, given that many diagnoses and assessments are derived from observation, and that physical exams are not always necessary for diagnosis and treatment [7]. Telemedicine in psychiatry was initially employed to extend care to areas with low access, particularly rural areas, and has since undergone exponential expansion. Tele-psychiatry has been used both in the hospital and outpatient setting, as well as for individual and group settings. Increased access to psychiatric care via telehealth has resulted in improved treatment rates [7]. Like other specialties, both patients and physicians find convenience in telepsychiatry appointments. Telemedicine offers other benefits specific to psychiatry, allowing psychiatrists to observe the patient’s home environment during a virtual visit. The at-home telemedicine experience has provided patients, particularly younger ones, a more comfortable and thus productive psychiatric care experience.

Telehealth in Rehabilitation

**Figure 2:**
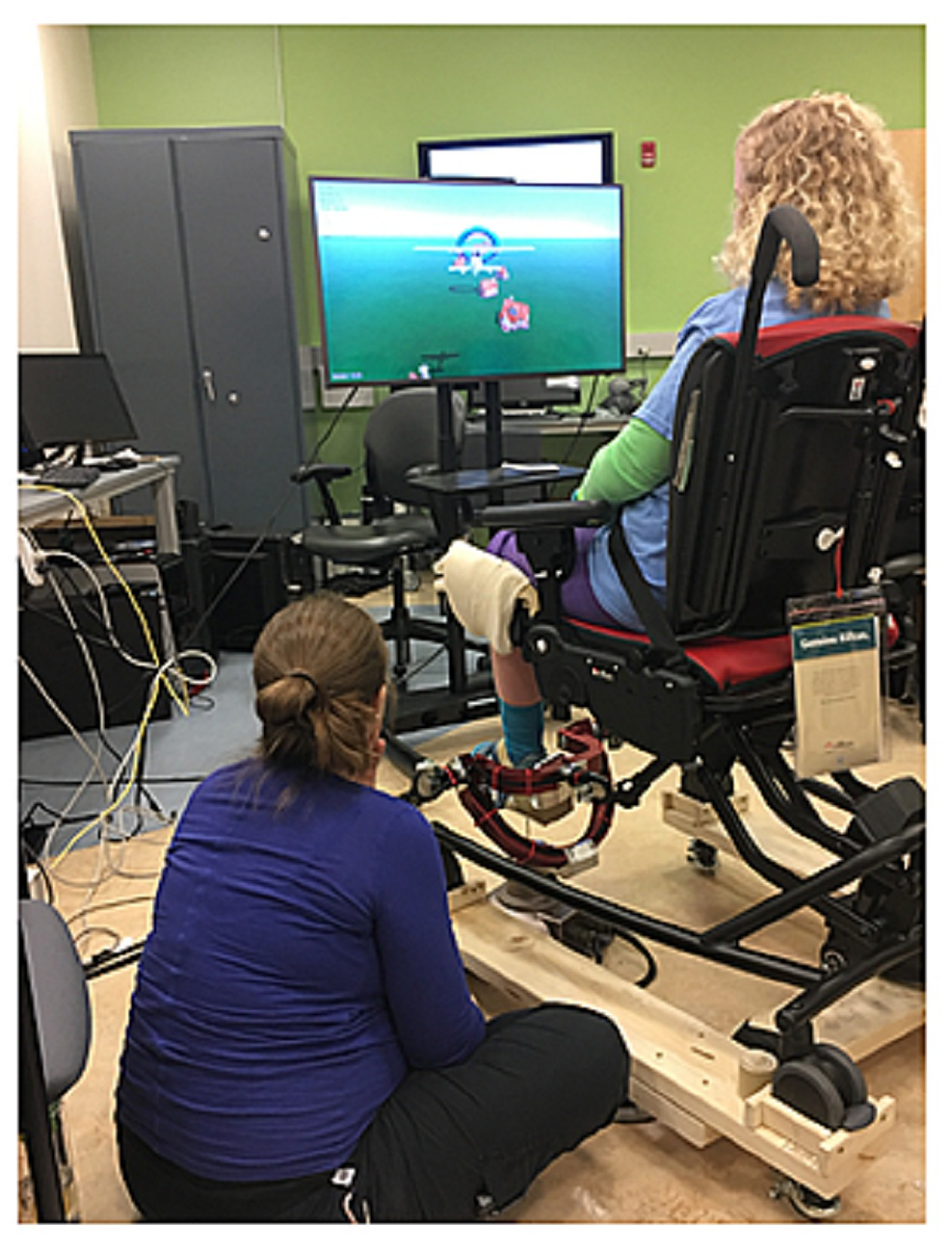
A pediatric patient demonstrating the use of a robotic rehabilitation system that incorporates gaming, which can be updated remotely to reflect rehabilitation goals Photo used with permission.

Rehabilitation, like many fields, has great potential for remote applications. Given the constraints of remote rehabilitation, ways to capture interest, especially in pediatric patients, warrants further investigation. For example, robotic rehabilitation has been applied to patients with cerebral palsy by combining it with video games (Figure 2) [8]. Children’s National Hospital reported the start of an at-home robotic rehabilitation program in the fall of 2020. The gaming tasks were updated each week remotely by the physical therapist based on the patient's progress. Convenience and ease of access to equipment in the home were highlighted by patients and families. This also highlights the key limitation of rehabilitation virtually, which is the lack of specialized equipment to distribute among patients in need. However, if this limitation is addressed, efficacy is similar to in-person therapy resulting from ease of access.

Clinic of the future

The use of telemedicine for outpatient visits has increased dramatically over the past year. Many areas of the ambulatory visit are ripe for innovation, from workflow, exam room design, physical exam, remote physical exam, EMR integration, incorporation of wearable technologies, ancillary services, and informatics [2]. Here, we discuss the virtual physical exam, wearable technology in primary care, and the future of the modern orthopedic clinic.

Virtual Physical Exam

The clinic of the future will rely heavily on technology to improve the experience for physicians and patients. Current telemedicine protocols are now operational, with institutions seeing patients at higher than pre-pandemic levels [6]. Virtual visits are limited by the physical exam that can be performed, creating an opening for innovation to improve the virtual visit and provide a more comprehensive experience for the physician. Establishing physical exam capabilities that mirror an in-person visit will increase the utility of telemedicine and increase the capacity of different specialties and service lines to provide virtual care. Opportunities for collaboration among specialties emerge given that a comprehensive virtual exam will likely include smartphone technology, photographic technology, and digital instruments with wireless capability.

Wearable Technology in Clinical Care

Wearable technologies are now an accepted and growing part of modern practice for both medical and surgical specialties [9]. The use of these devices ranges from primary care to medical and surgical subspecialties, including cardiology, neurology, rehabilitation, oncology, orthopedics, and neurosurgery [10-14]. Derived from cell phone technology, smartwatches, and other mobile devices, wearables in these fields have made remote monitoring possible. Data can now be digitalized, recorded, and transmitted from pulse oximeters, cardiac rhythm monitors, continuous glucose monitors, otoscopes, and devices for at-home sleep studies.

Moreover, both the types of data to be gathered and the number of devices available is rapidly expanding. It has even been proposed that wearable technology could replace the physical exam in some patients given the continuous, real-time, objective measurements of physiology and patient activities, which could offer increased sensitivity to changes over short-term intervals [14]. As devices miniaturize and their capabilities mature, we will likely continue to observe the transition from center-based care to patient-based care as analysis of continuously available patient data becomes more widely available between physician visits [14]. Industry partners, such as those developing apps and add-ons for mobile phones, will be key participants to support the acquisition, data mining, and data analysis of patient information.

Future of the Orthopedic Surgery Clinic

While virtual visits have seen rapid changes, so too have modernized clinics, which were discussed in the context of orthopedic surgery during our symposium. The opportunity exists for numerous innovations that could streamline clinic efficiency in orthopedics and provide convenience to physicians and patients. Areas of the orthopedic clinic that would benefit from innovation include the thorough physical exam, movement analysis, imaging, and casting.

Many decisions in orthopedic practice, such as indications for surgery and rehabilitation progression, rely on precise measurements from physical exams and imaging. However, most physical exams generally continue to rely on manual goniometers or solely on visual inspection. Digitizing this process would provide increased precision in measurements, thereby better informing surgical decision-making and standardizing research efforts. This could be accomplished with a mobile phone-based application that measures angles or a digital goniometer integrated into the EMR for accurate recording. This also would allow better comparisons of relevant measurements, such as joint angles between appointments, and provide high-quality patient records for clinical research and machine learning algorithms. A digital goniometer in app form would also be amenable for use in telemedicine. 

Secondly, gait analysis is a commonly performed orthopedic assessment that is a systematic analysis of human walking. It is often performed by visual observation. More complex patients require additional visits to a specific gait analysis laboratory for more in-depth assessments. Observation alone lacks precision and has significant variability between observers. In the clinic of the future, gait analysis hardware and software could be integrated into the floors and walls of the clinic. As patients go to a clinic room, their gait can be recorded and integrated into the EMR immediately ready for physician analysis. Furthermore, specialized pedobarometers could be integrated into the clinic floor to measure pressure across the plantar surface of the foot to characterize both static and dynamic pressures further. Likewise, a series of sensors with accelerometers, cameras, and data capturing and transmitting devices could be used for gait analysis via telemedicine.

Performing diagnostic imaging in the orthopedic clinic is often a bottleneck to overall clinic efficiency given the relative paucity of scanners compared to the number of practicing physicians. Ideally, each individual clinic room in the future would be equipped with its own low-dose X-ray machine. As machines continue to miniaturize, this will become more feasible. Additionally, ultrasound has recently become a more commonly used tool, especially for local injections among orthopedic surgeons. Handheld ultrasound machines with EMR integration would further foster convenience for the patient and physician. These would increase the clinic's efficiency and allow more in-office procedures to be performed, thus further optimizing clinic efficiency and cutting down unnecessary appointments. Ultrasound is also the most mobile of imaging technologies and is therefore very amenable to field use and mobile clinics.

Finally, innovations to casting would be a significant contribution to all orthopedic clinics. Since the development of fiberglass casts in the 1970s, few improvements have emerged, leaving room for innovation in this realm. Novel materials that maintain rigidity yet offer improved padding, waterproofing, and the ability to be easily adjusted would provide significantly improve patient quality of life. Additionally, dynamic fracture manipulation, which is the ability to precisely modulate a cast after it has been placed is not a capability among current casting materials. A casting material with this property would allow a cast to be manipulated to maintain the fracture alignment as the swelling surrounding the fracture decreases. Progressive manipulation of a cast in a predictable manner would significantly benefit deformity and club foot cases. Additionally, three-dimensional (3-D) scanning and 3-D printed casts would allow precision casting for personalized care. Plastic 3D-printed casts would allow the cast and the skin to be washed, reducing sores and allowing the skin to be cleaned. As the cost of 3-D printing declines, this technology is likely to become more prominent in orthopedic clinics.

## Discussion

In this paper, we present the proceedings of a symposium on “The Clinic of the Future and Telemedicine” from December 2020 (Table 1). We explored the developing trends from ongoing innovation to those spurred on by the COVID-19 pandemic. We reviewed the integration of telemedicine and wearable technology into modern clinical care. We delved into remote patient monitoring and patient evaluation capabilities through telehealth in the fields of genetics and metabolism, psychiatry, and rehabilitation medicine. We discussed the “Clinic of the Future” in the context of genetics and metabolism, orthopedic surgery, and primary care. We highlighted potential innovations in the future orthopedic clinic, encompassing the physical exam, gait analysis, imaging, and casting. Telemedicine has been rapidly adopted in a short period in nearly all fields of medicine; yet room for innovation still exists as remote exams, assessments, and monitoring are optimized.

**Table 1:** Main points from the symposium are summarized here, shown by section and medical specialty lecture EMR: electronic medical record

Clinic of the Future		Telehealth	
Specialty	Main Points	Specialty	Main Points
Genetics & Metabolism	•Improved remote physical exam offers increased access to care and medically fragile patients to remain home limiting travel •Seeks to develop a remote physical exam kit to enhance the utility of virtual visits	Genetics & Metabolism	•Seeks to develop an app & mobile phone-based photogrammetry technology dysmorphology exams •Would allow standardizing assessments during remote visits and remote exams.
Orthopedic Surgery	•EMR integrated physical exam instruments •In clinic movement analysis •In room imaging systems for more office-based procedures •Novel casting material for custom casts, increased patient comfort, and dynamic fracture manipulation	Psychiatry	•Amenable to virtual visits •Increases access to care •Allows greater insight into patient’s home and surroundings contributing to patient assessment •Equal diagnostic accuracy compared to in-person evaluation
Primary Care	•Wearable technology for continuous remote monitoring •Shifts patient care from a center-based system to a patient-based model	Tele-rehabilitation	•In home robotic rehabilitation systems with remote updating and gaming integration

National and international governing bodies of medical and surgical specialties have provided new guidelines for triaging and prioritizing visits to be virtual or in-person [1]. Telemedicine is a rapidly evolving field with the highest use among radiologists, psychiatrists, and cardiologists at reported rates of 39.5%, 27.8%, and 24.1% [5]. Given the pandemic, its use among other medical departments is rising, as reflected by all 50 states and Washington, DC, providing some form of reimbursement for telemedicine as of February 2020 [5]. Telemedicine in the United States is outpacing other countries, including the European Union, Korea, and Japan [5,15]. Telemedicine can be deployed in many formats and offers widespread utility to both patients and providers including “store and forward” (referring to the transmission of prerecorded data), live video consultations, and mobile health, which includes mobile apps, text messaging systems, and wearable technology [16].

Telemedicine has been implemented for several unique cases as a result of the COVID-19 pandemic. Within the emergency department (ED), telemedicine has been utilized as a “forward triage” service before a complete triage upon to the ED during the COVID-19 pandemic [5]. Telemedicine not only decreases the wait time to see a physician but also protects healthcare workers from exposure to COVID-19 [5]. In March 2020, the ED at Children’s National Hospital rapidly established a COVID-19 telemedicine clinic for follow-up virtual visits for patients deemed stable for discharge after coronavirus testing.

Telemedicine has been successfully used in both medical and surgical specialties. While telemedicine consults were originally largely used for outpatient and triage services, now in-patient consults have been reported to be performed via telemedicine [17]. Additionally, postoperative wound checks, complications, and pain management are well-suited to remote monitoring with text messaging and other applications [5]. Mobile health and wearables are to be significantly useful and the daily average step count has been correlated with good outcomes in spine surgery. Through the three speakers from different fields on our agenda, we explored the utility, applications, and future directions of the role of telemedicine.

Many advantages of telemedicine were shared across the medical specialties discussed in the symposium. Patients have improved follow-up visitations, reduced travel, more comfort from being home, reduced exposure, and efficient visits [18]. Access to care was also uniformly reported to be increased for both new and existing patients. Digital appointments circumvented traditional barriers to care such as distance to hospitals, clinic size, department workforce, and insurance coverage [6]. The greater access to care was also spurred on by time savings in many avenues. Patients saved on time for transportation and wait time, missed fewer workdays, and school days [18].

Providers, likewise, have seen many, benefits including improved follow-up and fewer missed appointments with clinical volumes equal to pre-pandemic levels [6]. Time savings through digital intake forms has allowed increased revenue given their ability to see more patients despite the per-patient reimbursement being lower due to reduced facility fees [5,16].

Additionally, both patients and physicians reported an increased convenience with some reporting a preference over traditional appointments. A noteworthy benefit to the medical system is also a reduced hospitalization rate [7,19]. Exposure to COVID-19 and other diseases is also minimized for both physician and patient thus reducing transmission risk. Finally, payers can insure more patients, extend coverage to underserved areas, and aggregate patient data on a larger scale [16].

While the field of telemedicine and individual specialties have appreciated both the shared and unique benefits of telemedicine, they also share limitations such as access to devices and Internet services. Additionally, concerns over licensure are now at the forefront of discussion. Now that virtual care can be easily provided across state lines, license and reimbursement policies will need to adapt to the changing doctor-patient relationship. Privacy is also a concern for both, parties given the sensitive nature of many health conditions and HIPAA. Screen burnout, fatigue, and distraction are all concerns that can impede a digital session for doctor and patient and are concerns to be considered for any telemedicine program. 

Psychiatry practice has found no difference in diagnostic accuracy or patient satisfaction between assessments performed in person versus telepsychiatry [7]. Likewise, treatment has been equally effective in delivering psychotherapy for anxiety disorders such as generalized anxiety disorder, post-traumatic stress disorder, and depression [7,20]. The growing trend of remote psychiatric care indicates that both patients and clinicians have an increasingly positive perception of the future of telemedicine in psychiatry [7]. Several limitations are a concern, however. While some may think a physical exam is unnecessary for psychiatric diagnosis, many disagree, especially when evaluating patients with substance withdrawal, agitation, or stupor [7]. Moreover, concerns include the difficulty of establishing a sound therapeutic alliance if a physician and patient had not previously met in person [7]. Long-term outcomes with telemedicine remain unknown in the field. Likewise, remote communication may not allow contextual clues to be observed that may be easily perceived in person but not during a live video session [7].

Telerehabilitation with robotic systems, in particular, has been increasing in use and is amenable to telemedicine. This sentiment is recognized in the literature and has demonstrated that tele-rehab is beneficial for reducing pain and improving physical function instead of face-to-face interaction [19]. Additional limitations similarly include lacking a thorough physical exam. Palpation, specific testing of muscles, and examining joint articulation remain unanswered challenges in tele-rehab [19]. Motion sensors and body monitoring technologies that quantify motion with contactless data retrieval have been proposed and may be integrated into rehab and tele-rehab programs [19]. Furthermore, virtual reality systems and haptic technology interfaces are emerging in parallel with rehab robotics and rehab gaming, as mentioned [19]. These further expand the rehab gaming sector, now known as “exergames,” which increases engagement in rehabilitation programs [19]. Emerging technologies and novel collaborations are trends worth following in the growing at-home rehabilitation sector. Virtual tele-rehab can offer care on a larger scale and likewise, increase access to care.

While telemedicine’s numerous benefits have been explored, a complete remote physical exam would increase the utility of virtual visits. App-based photogrammetric technology is being developed to aid in the assessment of growth parameters in pediatric patients. This could be used by patient families and sent via mobile app to the physician to provide uniform measurements that can be tracked over time. To further supplement innovation in virtual physical exams, a remote exam kit could be developed that patients could receive before appointments, which could either be kept by the patient for future visits or returned for use by another patient. Included in the kit could be devices for recording and transmitting vital signs and pertinent physical exam findings. The development of virtual physical exam kits will improve the usefulness of remote exams and continue to drive the field towards the clinic of the future.

Despite the high expectations for wearable technologies, several hurdles remain before implementation can achieve its full potential. Given the continuous nature of data collection, analysis, storage, security, and determining clinically relevant data remain challenges. While devices can be developed for collecting and transmitting data, methods of analysis that make it clinically meaningful must be developed in parallel. Machine learning algorithms have emerged as useful analytical methods and will likely remain integral to the field of big data. A potential limitation to the success of wearable technology in health care is patient cooperation. This is a challenge for physicians and product designers and engineers who are tasked with developing products that can be worn without discomfort. Nevertheless, the growing use of wearable technologies may drive a shift in care toward remote patient monitoring and the clinic of the future.

The orthopedic clinic of the future was explored here, citing four areas subject to innovation: the precise physical exam, movement analysis, imaging, and casting. Instruments that integrate into EMRs could provide precision, reliability, and accurate recording that minimizes error. Digital goniometers in app form could allow similar accuracy virtual appointments by similarly transmitting data through app and mobile phone technology. These tools could also standardize angle measuring across the field for increased reliability and decreased variation among providers. Additionally, the further integration of ancillary services into the clinic, such as gait analysis and imaging, would enhance the efficiency of an ambulatory clinic by reducing secondary appointments, better utilizing time, and allow more in-office procedures with minimal waiting time. Lastly, innovations to casting technology would further drive the field towards the clinic of the future. Novel materials and designs that allow dynamic fracture manipulation would allow changes to a patient’s cast to be made through time to optimize the treatment plan. Patient experience might also be improved as 3D-printed casts allow better hygiene and comfort.

## Conclusions

Here, we summarize the pertinent highlights from a symposium on the “The Clinic of the Future and Telehealth,” hosted by the National Children’s Health System in December 2020. Six speakers from five medical specialties discussed the ongoing trends of telemedicine pertinent to their fields and developing technologies that are ushering in the clinic of the future. A part of this shift in patient-centered care models is a change from center-based care to patient-centered care driven by remote patient monitoring, wearable technology, and the need for remote physical exams. As we continue in this age of medical innovation, significant opportunity exists to shape further the landscape of the emerging fields of telehealth and the clinic of the future.
